# Training programmes for healthcare professionals in managing epidural analgesia: A scoping review

**DOI:** 10.1111/aas.70025

**Published:** 2025-03-15

**Authors:** Cornelia Charlotte Lamprecht, Morten Vester‐Andersen, Thordis Thomsen, Tanja Eg Thomsen, Anne Mørup‐Petersen, Kim Wildgaard

**Affiliations:** ^1^ Department of Orthopaedic Surgery Copenhagen University Hospital, Herlev and Gentofte Hospital Herlev Denmark; ^2^ Herlev Anaesthesia, Critical and Emergency Care Science Unit (ACES), Department of Anaesthesiology and Intensive Care Medicine Copenhagen University Hospital, Herlev and Gentofte Hospital Herlev Denmark; ^3^ Department of Clinical Medicine, Faculty of Health Sciences University of Copenhagen Copenhagen Denmark

**Keywords:** clinical education, educational interventions, epidural analgesia, healthcare professionals, pain management, training programmes

## Abstract

**Background:**

Epidural analgesia (EA) is widely used for postoperative and labour pain management. Systematic training of healthcare professionals, particularly nurses, is essential for the safe administration and management. This scoping review aimed to identify and map existing EA training programmes.

**Methods:**

A PRISMA‐ScR‐guided search was conducted across multiple databases and grey literature. Studies on educational interventions for healthcare professionals in EA management were included. Data extraction and categorisation were performed using Kirkpatrick's Four‐Level Training Evaluation Model.

**Results:**

Eighteen studies were included, covering classroom training, workshops, self‐directed learning, simulation‐based training, and on‐the‐job training. Participants were primarily nurses. Programmes addressed epidural techniques, monitoring and assessment, spinal anatomy and pharmacology, complication management, and patient care. Most studies focused on short‐term knowledge gains, with a limited assessment of long‐term clinical impacts.

**Conclusion:**

Limited research exists on EA training, with most programmes targeting nurses and relying on classroom‐based training. Training structures varied, and evaluations primarily assessed short‐term knowledge gains.

**Editorial Comment:**

The authors conducted a literature search to get an overview of programmes that aimed to train healthcare staff in managing epidural pain relief. Most of the identified 18 studies described classroom teaching and focused primarily on knowledge about complications. Patient contact in this type of training was not reported. The effectiveness of this type of training in a clinical context was difficult to evaluate based on the published evidence.

## 1 INTRODUCTION

Epidural analgesia (EA) is widely used in postoperative and labour settings for effective pain management. It enhances patient comfort, accelerates recovery, and reduces complications, including chronic postoperative pain.^1^, ^2^ Across various surgical populations, EA offers substantial benefits such as improved physical function, reduced nausea and sedation, and superior recovery outcomes.^2^, ^3^ Additionally, EA reduces postoperative respiratory complications and delivers more consistent and superior pain relief compared with systemic opioid treatment.^3^, ^4^

Despite advancements in pain management, severe postoperative pain remains common and is often underestimated, leading to negative outcomes such as delayed recovery, prolonged hospital stays, and increased opioid reliance.^2^, ^5^, ^6^, ^7^, ^8^

For this review, “management of epidurals” refers to the set of activities required to ensure safe and effective pain relief through EA. After initial dosing and adjustments by anaesthetists, this includes patient monitoring, adherence to clinical protocols, early detection and management of complications and dose adjustments when patients experience breakthrough pain, and timely communication among care teams. Effective EA management requires an interdisciplinary approach.^9^ Effective communication and timely escalation of care are essential but increasing demands on healthcare systems have shifted responsibilities from specialised teams, such as Acute Pain Services (APS), to ward nurses. Without sufficient training and support, this shift increases the risk of adverse events, delayed complication recognition, and poor pain control. Ensuring high‐quality training for nurses is key to maintaining patient safety and reducing these risks.^9^, ^10^, ^11^, ^12^, ^13^

Despite their critical role, nurses often lack the knowledge and skills necessary for EA management.^14^ Studies highlight gaps in nurses' understanding of key areas such as spinal cord anatomy, pharmacology of epidural medications, complication management, and adherence to clinical guidelines.^15^, ^16^, ^17^ EA is a complex pain management technique requiring specialised training in patient monitoring, protocol adherence, and the safe operation of medical equipment.^2^, ^16^, ^17^ Without adequate education, healthcare professionals may fail to detect complications early, leading to poor pain control and avoidable patient harm.^2^, ^8^

Given the increasing reliance on nurses in EA management and the risks posed by inadequate training, this scoping review aims to systematically identify and map existing training programmes for healthcare professionals. By highlighting gaps and opportunities, this review seeks to guide future efforts in improving training, ensuring safe EA management, and optimising patient outcomes.

## 2 METHODS

This scoping review followed the guidelines outlined in the Preferred Reporting Items for Systematic Reviews and Meta‐Analyses extension for Scoping Reviews (PRISMA‐ScR),^18^, ^19^ and utilised the framework developed by Arksey and O'Malley, later refined by Levac.^20^, ^21^ The review was conducted in accordance with our pre‐published protocol,^19^ with minor amendments detailed in the supplementary information, including a list of changes.

### 2.1 Review questions

- *What training programmes have been used to train healthcare professionals in managing epidural analgesia in clinical settings?*
  - *Who were the healthcare professionals attending the training programmes?*
  - *Who was responsible for the development and delivery of the training programmes?*
  - *What was the content and duration of the training programmes?*
  - *How was the effectiveness of the training programmes measured?*

### 2.2 Inclusion criteria

We included all study types that focused on educational interventions aimed at healthcare professionals to safely manage EA. Studies published in English, German, Danish, Swedish, and Norwegian were eligible. We excluded studies that exclusively addressed epidural catheter insertion or intraoperative use, as well as procedural articles that did not include an evaluation of a training programme.

### 2.3 Search strategy

A systematic search was performed in August 2023 and updated in November 2024 in MEDLINE/PubMed, CINAHL, EMBASE, ERIC, and the Cochrane Central Register of Controlled Trials (CENTRAL). We also explored unpublished or grey literature through Mednar, WorldWideScience.org, and OAlster. Additionally, manual searches and citation tracking were conducted. The search strategy was developed using the Population, Concept, and Context framework.^22^ The final search string is provided in Data S1.

### 2.4 Study selection

All identified citations were uploaded into the Covidence Systematic Review Software,^23^ where duplicates were removed. Titles and abstracts were screened, and full‐text studies were independently assessed by two authors (CL/TET). To ensure consistency, the review team met at the beginning, midpoint, and final stages of the abstract review process to address any methodological challenges or uncertainties.^21^ Any disagreements were resolved by a third researcher (TT), serving as an arbitrator.

During the full‐text review phase, we contacted authors, searched institutional repositories, and requested interlibrary loans to obtain all relevant studies. However, four reports remained inaccessible.

### 2.5 Data extraction

A data charting form was developed in Covidence^23^ to extract the following information: title, author, year, country, aim, study design, participants, type of educational intervention, developer (organisation or individual(s) responsible for creating the intervention), facilitator(s) (person or team delivering the intervention), content, duration of the educational intervention, and outcome measures. Two authors (CL, TT) independently extracted data from the first five included studies and compared their results to ensure accuracy and reliability.^21^ Subsequently, one author (CL) continued the data extraction for the remaining studies, with regular checks by the second author to maintain consistency.

### 2.6 Data synthesis and analysis

We used an iterative, inductive approach to thematically organise data from the included studies. Extracted information was repeatedly analysed to identify recurring patterns, similarities, and differences in educational practices for managing EA.

The outcome measures used in the included studies were categorised using Kirkpatrick's Four‐Level Training Evaluation Model, which assesses training effectiveness across four levels.^24^ The model evaluates immediate outcomes such as satisfaction and knowledge acquisition (Levels 1 and 2), the application of learned skills and professional competency in the workplace (Level 3), and broader impacts on organisational or clinical outcomes (Level 4).^24^, ^25^

Consensus on the final groupings was reached by all authors.

## 3 RESULTS

**FIGURE 1:**
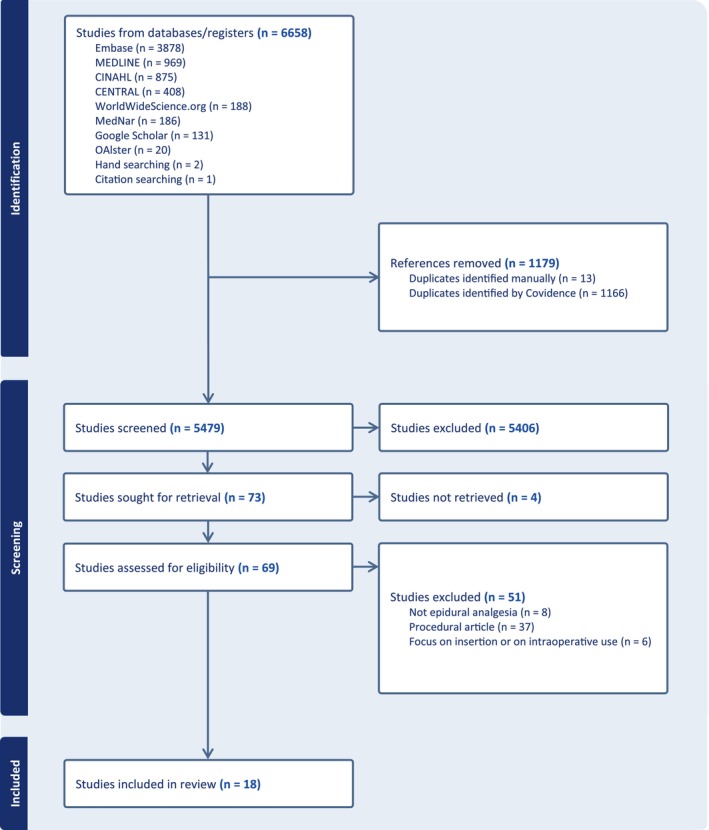
PRISMA flow chart of study selection: training programmes in epidural analgesia management.

Eighteen studies were included in this review. We refer to Figure 1 for the study selection process.

### 3.1 Study characteristics

The studies employed pre‐/post‐test^26^, ^27^, ^28^, ^29^, ^30^, ^31^, ^32^, ^33^, ^34^, ^35^, ^36^, ^37^, ^38^, ^39^ and descriptive designs.^40^, ^41^, ^42^, ^43^ The majority were conducted in the United States,^26^, ^28^, ^32^, ^35^, ^36^, ^38^, ^39^, ^40^ followed by Canada,^30^, ^31^, ^34^, ^42^ and the United Kingdom.^33^, ^37^, ^39^ The studies were published from 1990 to 2023, with the highest concentration from 2011 to 2020. Participants were primarily nurses.^26^, ^27^, ^28^, ^30^, ^31^, ^32^, ^33^, ^34^, ^35^, ^37^, ^38^, ^39^, ^40^, ^41^, ^42^, ^43^ Physicians participated in six studies,^29^, ^35^, ^36^, ^37^, ^42^, ^43^ while midwives were included in one,^34^ and pharmacists in another study.^43^ Table 1 provides a summary of key characteristics.

**TABLE 1:** General study characteristics.

Characteristics	*n*	%
Study design		
Pre‐/Post‐test design	14	77.7
Descriptive design	4	22.3
Country		
USA	8	44.4
Canada	4	22.2
UK	3	16.7
Australia	1	5.6
Japan	1	5.6
Argentina	1	5.6
Year of publication		
1990–2000	4	22.2
2001–2010	4	22.2
2011–2020	8	44.4
2021–2023	2	11.1
Participants		
Nurses	16	88.9
Physicians	6	33.3
Midwives	1	5.6
Pharmacists	1	5.6

### 3.2 Types of training programmes

The studies utilised five main training modalities: classroom training, workshops, self‐directed training, simulation‐based training, and on‐the‐job training. Additionally, 11 training programmes incorporated standardised procedures, such as policies, guidelines, and protocols.^26^, ^30^, ^31^, ^34^, ^35^, ^36^, ^38^, ^39^, ^40^, ^41^, ^43^ Classroom training was the most frequently used modality.

**FIGURE 2:**
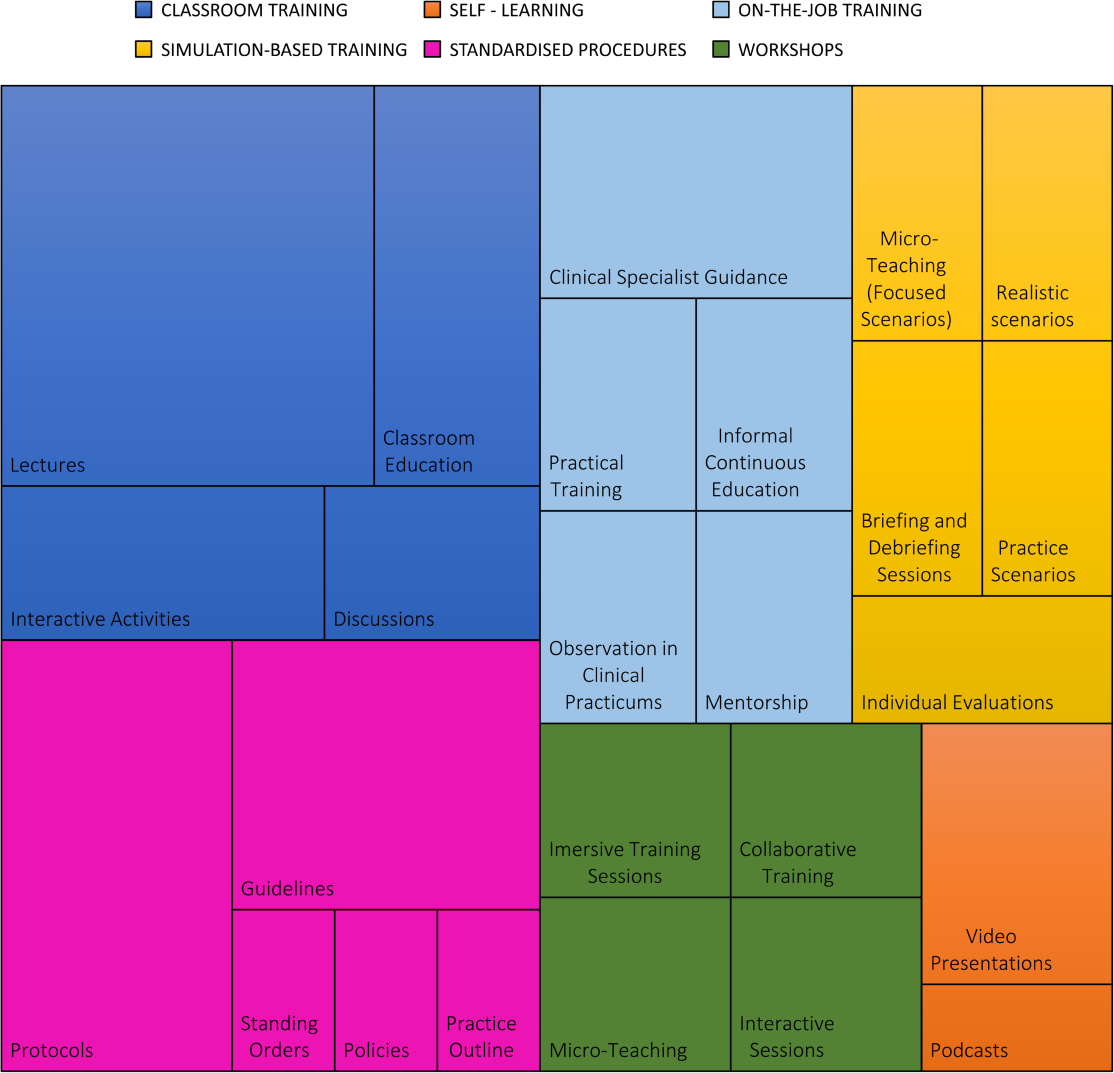
Didactic approaches used within the different training modalities. The rectangle sizes reflect the frequency of each component's use in the included studies.

Table 2 provides an overview of training modalities and supporting standardised procedures in the included studies, while Figure 2 outlines the didactic approaches used across different training modalities.

**TABLE 2:** Use of training and supporting standardised procedures in included studies.

First author	Country	Year	Classroom training	Self‐directed training	On‐the‐job training	Workshops	Simulation‐based training	Standardised procedures^a^
Camp‐Sorell	USA	1990	✓		✓			✓
Anderson‐Estill	USA	1993	✓		✓			✓
Nowakowski	USA	1995	✓		✓			✓
O'Brian	USA	1995		✓				
Bibby	Australia	2001	✓					✓
Richardson	USA	2001	✓		✓			✓
Ellis	Canada	2007	✓			✓	✓	
Ingelmo	Argentina	2007	✓		✓			✓
Haas	USA	2015	✓					
O'Connor	UK	2015	✓					
Luctar‐Flude	Canada	2018	✓	✓	✓	✓	✓	✓
Sawhney	Canada	2018	✓		✓	✓	✓	✓
Kariya	Japan	2020					✓	
Sawhney	Canada	2020	✓		✓	✓	✓	✓
Zheng	USA	2020						✓
Cook	UK	2020	✓		✓			
Puthoff	USA	2021	✓		✓			✓
Kameni	USA	2023		✓				

^a^ Standardised procedures, for example, policies, guidelines, protocols.

#### 3.2.1 Classroom training

Fourteen studies described structured theoretical teaching in a traditional classroom setting, with direct interaction and immediate feedback.^26^, ^27^, ^30^, ^31^, ^33^, ^34^, ^35^, ^37^, ^38^, ^39^, ^40^, ^41^, ^42^, ^43^

#### 3.2.2 Workshops

Four studies combined theoretical learning with practical skill development, emphasising hands‐on practice.^30^, ^31^, ^34^, ^42^

#### 3.2.3 Self‐learning

One study implemented a self‐learning packet as material for independent self‐study.^32^ Two studies used online learning via digital platforms to deliver theoretical knowledge.^28^, ^34^

#### 3.2.4 Simulation‐based training

Five studies employed simulation‐based training, allowing participants to practice in realistic, controlled scenarios that mirror clinical situations.^29^, ^30^, ^31^, ^34^, ^42^

#### 3.2.5 On‐the‐job training

Eight studies offered hands‐on clinical training with real‐time guidance, typically involving a supervisor or mentor providing immediate feedback and support during clinical procedures.^26^, ^34^, ^35^, ^37^, ^38^, ^39^, ^40^, ^43^

#### 3.2.6 Standardised procedures

Eleven studies included standardised procedures as a supporting method to ensure consistent practice and facilitate implementation.^26^, ^30^, ^31^, ^35^, ^36^, ^38^, ^39^, ^40^, ^41^, ^42^, ^43^

### 3.3 Training programmes development and organisation

The training programmes were developed by diverse teams, including anaesthesia departments,^40^ multidisciplinary teams (MDTs),^26^, ^30^, ^31^, ^37^, ^39^, ^41^ interdepartmental teams,^38^, ^42^, ^43^ and research teams.^28^, ^29^, ^33^, ^34^, ^35^, ^36^ The training programmes were delivered by clinical nurse specialists,^26^, ^32^, ^37^, ^38^, ^39^, ^42^ nurse anaesthetists, and anaesthetists,^37^, ^39^, ^40^, ^42^, ^43^ expert nurses from the Acute Pain Service,^26^, ^30^, ^31^, ^32^, ^41^ and research teams.^33^, ^34^, ^35^, ^36^

### 3.4 Content of training programmes

**FIGURE 3:**
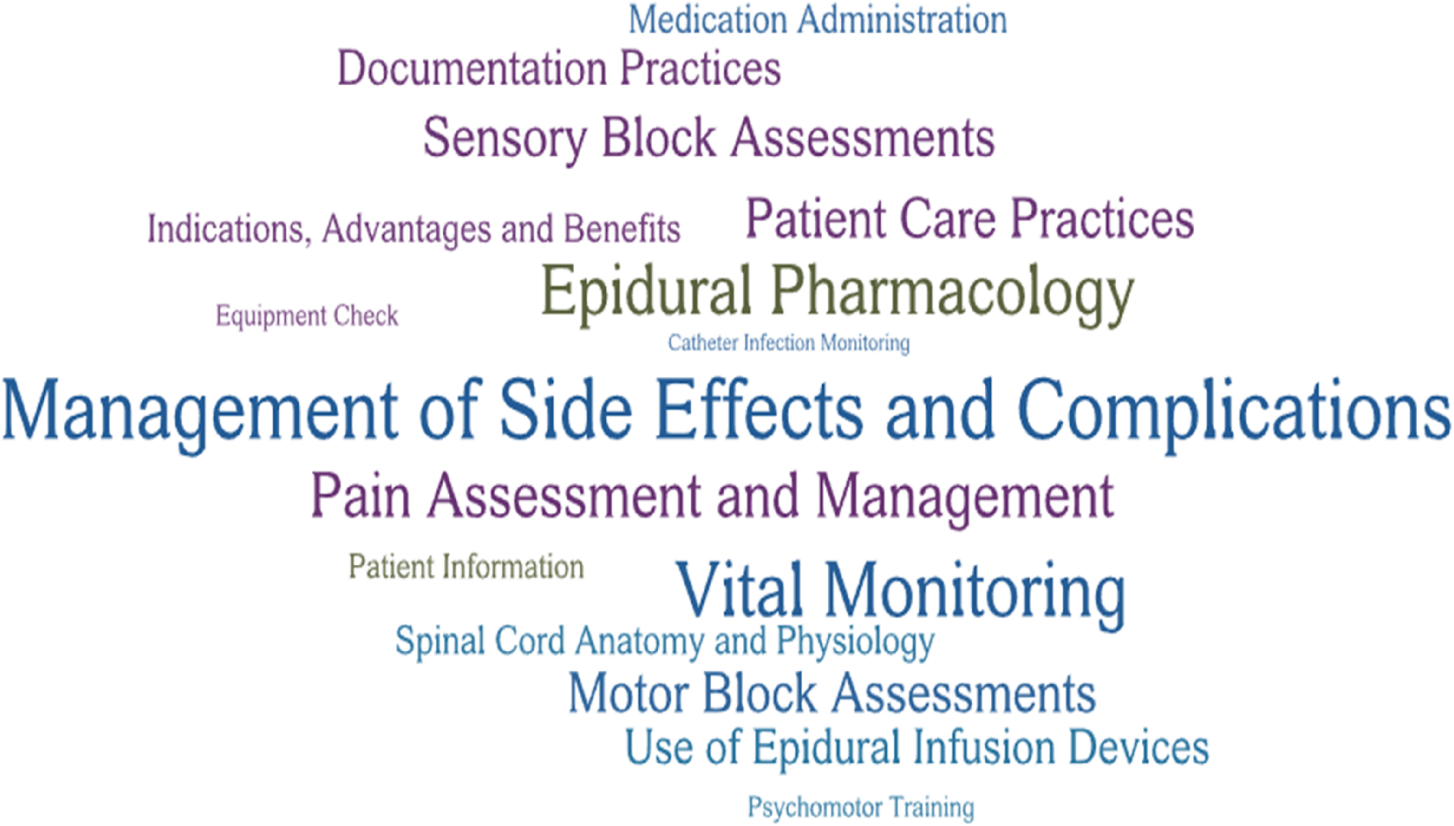
Key topics in training programmes—font size reflects the frequency of topics addressed in the training programmes.

The curricula in the training programmes addressed five core domains: epidural techniques and management, monitoring and assessment, pharmacology and physiology, complications and emergency management, and patient care and education. Refer to Figure 3 for the depiction of key topics in training curricula.

#### 3.4.1 Epidural techniques

Seven studies addressed the verification of the correct epidural catheter placement and removal,^26^, ^28^, ^32^, ^34^, ^35^, ^38^, ^39^ five studies included training on epidural infusion devices,^35^, ^38^, ^39^, ^41^, ^42^; two studies covered equipment checks,^30^, ^31^ and two focused on psychomotor training.^39^, ^42^

#### 3.4.2 Monitoring and assessment

Nine studies addressed vital monitoring,^26^, ^29^, ^30^, ^31^, ^32^, ^34^, ^36^, ^38^, ^42^ five studies focused on documentation practices,^34^, ^37^, ^38^, ^40^, ^42^ six studies covered motor block assessments,^30^, ^31^, ^32^, ^34^, ^37^, ^38^ six studies included sensory block assessments,^27^, ^30^, ^31^, ^32^, ^33^, ^34^ one study addressed catheter infection monitoring,^38^ and seven studies focused on pain assessment and management.^30^, ^31^, ^32^, ^34^, ^35^, ^38^, ^43^

#### 3.4.3 Spinal cord anatomy, physiology, and pharmacology

Five studies addressed spinal cord anatomy and physiology,^30^, ^31^, ^34^, ^40^, ^42^ eight focused on epidural pharmacology,^26^, ^30^, ^31^, ^32^, ^34^, ^35^, ^40^, ^42^ and five covered medication administration.^26^, ^29^, ^34^, ^35^, ^40^

#### 3.4.4 Complications and emergency management

Twelve studies highlighted the observation and management of side effects and complications.^26^, ^27^, ^28^, ^30^, ^31^, ^32^, ^33^, ^34^, ^35^, ^38^, ^39^, ^40^

#### 3.4.5 Patient care and education

Six studies addressed patient care practices,^30^, ^31^, ^32^, ^37^, ^38^, ^39^ three studies included patient information and education,^26^, ^32^, ^43^ and five studies focused on providing patients with information regarding the indications, advantages, and benefits of epidurals.^26^, ^32^, ^33^, ^35^, ^39^

### 3.5 Duration of training programmes

The duration of the included training programmes varied considerably. Four training programmes featured short sessions lasting between 30 min and 1 h of classroom teaching.^27^, ^30^, ^31^, ^40^ Workshops were reported to last 4 h.^26^, ^30^, ^31^, ^34^, ^42^ Self‐learners in one study had 2 months to complete the distance learning course.^28^ Seven studies did not specify the duration of their training interventions.^29^, ^33^, ^35^, ^36^, ^37^, ^38^, ^41^, ^43^

### 3.6 Outcome Measurement

**FIGURE 4:**
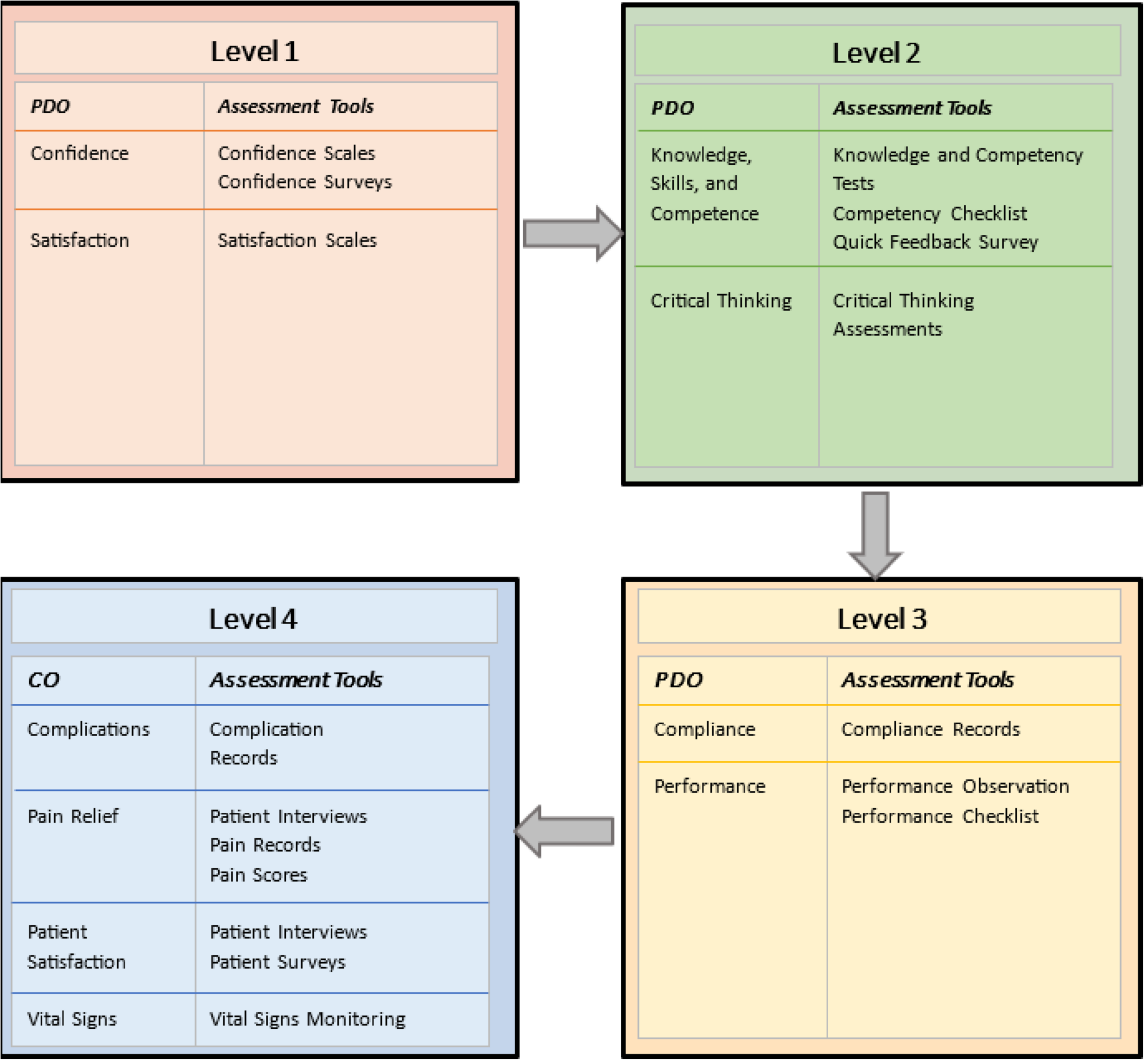
Overview of assessment levels and tools, categorising the tools used in the studies by their focus on professional development outcomes (PDO) and clinical outcomes (CO).

The effectiveness of the training programmes was measured using Kirkpatrick's four levels of evaluation, as described under Section 2.6. Immediate reactions after training, categorised as Level 1, were assessed in six studies. Short‐term outcomes focused on knowledge gain, representing Level 2, were evaluated in 15 studies.^26^, ^27^, ^28^, ^29^, ^30^, ^31^, ^32^, ^33^, ^34^, ^35^, ^36^, ^37^, ^38^, ^39^, ^40^ Intermediate changes in workplace behaviour, which align with Level 3, were explored in eight studies.^26^, ^35^, ^38^, ^39^, ^40^, ^42^, ^43^ Long‐term impacts on patient outcomes, classified as Level 4, were assessed in seven studies.^26^, ^35^, ^36^, ^38^, ^39^, ^40^, ^41^, ^42^, ^43^ Refer to Table 3 for evaluation levels used in the studies and Figure 4 for assessment tools used for the evaluation.

**TABLE 3:** Kirkpatrick levels addressed in included studies.

First author	Country	Year	Level 1 (reaction)	Level 2 (learning)	Level 3 (behaviour)	Level 4 (results)
Camp‐Sorell	USA	1990	✓	✓	✓	✓
Anderson‐Estill	USA	1993		✓	✓	✓
Nowakowski	USA	1995		✓	✓	✓
O'Brian	USA	1995		✓		
Bibby	Australia	2001				✓
Richardson	USA	2001	✓	✓	✓	✓
Ellis	Canada	2007			✓	✓
Ingelmo	Argentina	2007			✓	✓
Haas	USA	2015		✓		
O'Connor	UK	2015		✓		
Luctar‐Flude	Canada	2018	✓	✓		
Sawhney	Canada	2018	✓	✓		
Kariya	Japan	2020		✓		
Sawhney	Canada	2020	✓	✓		
Zheng	USA	2020		✓		
Cook	UK	2020		✓		
Puthoff	USA	2021		✓	✓	✓
Kameni	USA	2023	✓	✓		

## 4 DISCUSSION

In this scoping review, we identified 18 studies evaluating training programmes for managing EA. These programmes were developed and delivered by diverse teams of experts and researchers. Classroom training was the most frequently used modality, both as a standalone method and within blended learning approaches. Despite the increasing accessibility of digital tools, few studies incorporated online or simulation‐based training. The effectiveness of the training programmes was measured across all four levels of Kirkpatrick's evaluation model,^24^ with a primary focus on knowledge acquisition.^26^, ^27^, ^28^, ^29^, ^30^, ^31^, ^32^, ^33^, ^34^, ^35^, ^36^, ^37^, ^38^, ^39^, ^40^ These findings set the stage for discussing the implications, limitations, and opportunities for advancing EA training.

Although EA is widely used in various healthcare contexts,^1^, ^2^ we identified only 18 relevant studies primarily evaluating training programmes for nurses managing postoperative EA. Only one study included midwives alongside nurses in the EA training programme.^34^

The establishment of Acute Pain Services (APS) in larger hospitals in the late 20th century centralised the management of complex analgesic techniques under specialised teams. A primary reason for introducing APS was the limited experience of ward nurses in managing EA.^12^ With APS teams assuming responsibility for EA, the focus on developing ward nurses' competencies and practices in this area likely diminished over time. However, with increasing demands on healthcare systems and the need to optimise resource utilisation, there may be a growing emphasis on providing ward nurses with the necessary skills and knowledge to manage EA.^13^ Without adequate training and support, this transition poses a risk of delayed complication detection, suboptimal pain control, and potential adverse effects.

Involving multidisciplinary teams in programme development, as observed in six studies,^26^, ^30^, ^31^, ^37^, ^39^, ^41^ enhanced the integration of diverse expertise, improving the quality and effectiveness of training.^44^ However, patient involvement—recognised as critical for aligning educational content with patient needs and expectations^45^, ^46^, ^47^—was absent in all reviewed programmes. Including patient perspectives, such as experiences with pain management and mobility, could help create more patient‐centred and impactful training programmes.^45^, ^46^

Most training programmes employed blended learning,^26^, ^30^, ^31^, ^34^, ^35^, ^37^, ^38^, ^39^, ^40^, ^42^, ^43^ combining multiple pedagogical methods recognised as effective in healthcare education.^48^ Classroom teaching, however, remained the predominant approach, likely reflecting the period in which the studies were conducted. Despite the increasing availability after 2010, only two studies^28^, ^34^ incorporated digital solutions, possibly due to resource constraints or a preference for traditional methods.^24^, ^49^

Simulation‐based training consistently demonstrated superiority over traditional methods in enhancing skill retention and clinical competency. This approach closely aligns with Kirkpatrick's model by providing real‐world scenarios that enhance learning (Level 2), facilitate behavioural change (Level 3), and have the potential to improve patient outcomes (Level 4). It also addresses immediate reactions (Level 1) by building clinical competence and confidence in critical procedures. By fostering active engagement, refining skills, and encouraging reflection, simulation‐based training resonates with evidence that learners prefer activities grounded in clear theoretical concepts.^50^ Despite its proven effectiveness,^50^ simulation‐based training was employed in only five of the included studies.^29^, ^30^, ^31^, ^34^, ^42^ High costs and resource demand likely hinder broader adoption, as high‐fidelity simulations require specialised equipment, dedicated facilities, skilled instructors, and temporarily remove clinicians from patient care.^51^

The evaluation of training programme effectiveness in the included studies primarily focused on Level 2 of Kirkpatrick's model, which emphasises immediate knowledge and skill acquisition. While this focus is important, it overlooks other critical dimensions, such as participant satisfaction at Level 1, behavioural changes in clinical practice at Level 3, and long‐term impacts on patient outcomes at Level 4. Without addressing these additional levels, the assessment of training effectiveness remains incomplete.

Few studies explored Levels 3 and 4, which assess behavioural changes and clinical outcomes, including measures like patient satisfaction and complication rates. Future research should expand beyond short‐term knowledge gains to incorporate these dimensions. Greater emphasis on participant satisfaction at Level 1 is also essential, as it offers valuable insights into the acceptability and practicality of training programmes.

While knowledge acquisition is important, the true measure of success lies in the retention and application of this knowledge to improve patient care.^24^ A comprehensive evaluation spanning all levels of Kirkpatrick's model—participant satisfaction, knowledge acquisition, skill application, and patient outcomes—would offer a more holistic understanding of training impact. Including patient‐reported outcomes could further validate the results and strengthen the evaluation framework.

These findings underline the need for healthcare systems to embrace innovative training methods, such as digital and simulation‐based solutions, to address current gaps in EA training. Incorporating patient perspectives into programme design and evaluation aligns with global trends toward patient‐centred care^45^, ^46^, ^47^ and could enhance the relevance and effectiveness of training programmes. Furthermore, standardising the duration and structure of training interventions may facilitate better comparisons and improvements across different settings.

## 5 STRENGTHS AND LIMITATIONS

The main strengths of this scoping review include its broad and comprehensive search strategy, which covered multiple databases and grey literature. Adherence to the PRISMA‐ScR statement^18^ and the published pre‐study protocol^19^ further strengthens the rigour of the review.

Despite a thorough search strategy, some studies may have been overlooked due to language restrictions and database limitations. Most included studies originated from the United States, Canada, and the United Kingdom, limiting generalisability to other healthcare settings. Research scarcity may be attributed to the high resource demands of training programmes, underreporting of inconclusive results, and a focus on unpublished, locally adapted initiatives.^52^, ^53^ Additionally, competing research priorities and the integration of epidural training into broader curricula may have reduced the focus on standalone studies.^54^

Inconsistent reporting of training content, structure, and duration made systematic categorisation and comparison challenging. Additionally, many studies did not specify the trainers, limiting insights into the delivery and implementation of the training.

This scoping review did not assess the effectiveness of the training programmes. However, study design limitations affected the strength of the conclusions. Four studies^40^, ^41^, ^42^, ^43^ employed descriptive designs, which lack the analytical depth to establish causal relationships.^55^ The remaining 14 studies^26^, ^27^, ^28^, ^29^, ^31^, ^32^, ^33^, ^34^, ^35^, ^36^, ^37^, ^38^, ^39^, ^56^ used pre/post‐test designs, making it difficult to isolate the effects from confounding factors such as natural skill improvement over time or broader changes in clinical practice.^57^

## 6 CONCLUSION

This review identified 18 studies on epidural analgesia training, primarily targeting nurses and using classroom‐based training. Training structures varied, and evaluations mainly assessed short‐term knowledge gains, with limited focus on practical skills and long‐term competency.

## AUTHOR CONTRIBUTIONS

CL conceptualised the study, conducted screening, performed data charting, and drafted the manuscript. TET contributed to screening and provided revisions to the manuscript. MVA, TT, AMP, and KW participated in conceptualising the study and provided critical revisions through the final manuscript review.

## FUNDING INFORMATION

This work was supported by funding from the Department of Orthopaedic Surgery and the Department of Anaesthesiology at Herlev and Gentofte Hospital, Capital Region of Denmark.

## CONFLICT OF INTEREST STATEMENT

The authors declare no conflicts of interest.

## Supporting information


**Data S1.** Supporting Information.

## Data Availability

Data sharing is not applicable to this article as no new data were created or analyzed in this study.
