# Supplementary material for: Training programmes for healthcare professionals in managing epidural analgesia: A scoping review
Source: Acta Anaesthesiol Scand. 2025 Mar 15;69(4):e70025. doi: 10.1111/aas.70025 (PMC11912511; doi:10.1111/aas.70025)
Supplement: Supplementary file 1 — Data S1. Supporting Information. [file AAS-69-0-s001.docx]

# Supplementary Data

## Section 1: Protocol Amendments

| **Amendment’s Date** | **Original Protocol** | **Description of Amendment** |
| --- | --- | --- |
| 2024-06-25 | Title: “Training Programmes for Healthcare Professionals in Managing Postoperative Epidural Analgesia” | Revised title: “Training Programmes for Healthcare Professionals in Managing Epidural Analgesia” |
|  |  |  |
|  |  |  |

## Section 2: Search Strategi

**Database:**
Embase <1974 to 2023 September 27>

| # | Query | Results from 28 Sep 2023 |
| --- | --- | --- |
| 1 | ((healthcare adj1 professional*) or (health adj1 care adj1 professional*) or (healthcare adj1 personnel) or (health adj1 care adj1 personnel) or (healthcare adj1 staff) or (health adj1 care adj1 staff) or (healthcare adj1 worker*) or (health adj1 care adj1 worker*) or nurse* or nursing or midwif* or midwifes or physician* or doctor* or (medical adj1 staff) or (medical adj1 personnel)).mp. [mp=title, abstract, heading word, drug trade name, original title, device manufacturer, drug manufacturer, device trade name, keyword heading word, floating subheading word, candidate term word] | 2,121,684 |
| 2 | ((After adj1 operation*) or postoperative or post-operative or (post adj1 operative) or post-surgical or post-op or (after adj1 surgery) or perioperative).mp. [mp=title, abstract, heading word, drug trade name, original title, device manufacturer, drug manufacturer, device trade name, keyword heading word, floating subheading word, candidate term word] | 1,612,410 |
| 3 | ((epidural adj1 analgesia) or (epidural adj1 anaesthesia) or (epidural adj1 anesthesia) or (local adj1 analgesia) or (local adj1 anaesthesia) or (local adj1 anesthesia) or (regional adj1 analgesia) or (regional adj1 anaesthesia) or (local adj1 anesthesia)).mp. [mp=title, abstract, heading word, drug trade name, original title, device manufacturer, drug manufacturer, device trade name, keyword heading word, floating subheading word, candidate term word] | 99,966 |
| 4 | Epidural Analgesial/ | 0 |
| 5 | Epidural anesthesia/ | 32,244 |
| 6 | exp nursing/ or midwifery/ | 404,204 |
| 7 | exp health personnel/ | 1,981,865 |
| 8 | 6 or 7 | 2,262,106 |
| 9 | 1 or 8 | 3,018,414 |
| 10 | 3 or 4 or 5 | 99,966 |
| 11 | 9 and 10 | 11,554 |
| 12 | Postoperative Period/ or Postoperative Care/ or Postoperative Pain/ or Postoperative Complications/ or exp Perioperative Care/ | 616,836 |
| 13 | 2 or 12 | 1,640,074 |
| 14 | 11 and 13 | 4,096 |
| 15 | (teach* or train* or educ* or simulat* or curriculum or workshop*).mp. | 3,371,232 |
| 16 | 14 and 15 | 739 |
| 17 | limit 14 to human | 3,878 |
| 18 | limit 16 to human | 692 |

### Section 3: Study Characteristics

| **Author** | **Title** | **Year** | **Country** | **Aim** | **Study design** | **Participants** | **Type of educational intervention** | **Developer** | **Facilitator** | **Content** | **Duration** | **Outcome Measures** |
| --- | --- | --- | --- | --- | --- | --- | --- | --- | --- | --- | --- | --- |
| Anderson-Estill | Epidural and intrathecal analgesia in a rural setting. | 1993 | USA | Describing the initiation of a program for nursing-administered epidural analgesia | Descriptive | Nurses | Lecture, practical training, videocassette, protocol | Department of Anaesthesia | Anaesthesia staff, Nurses | Anatomy and physiology, opioid pharmacology, side effects, epidural policies | 50-min video,  1-hr session 30-min exam | Nurse competency, patient safety (monitoring complications), pain relief (patient reported pain levels), documentation accuracy  . |
| Bibby | Introducing ward-based epidural pain relief. | 2001 | Australia | Development and implementation of ward-based epidural service | Descriptive | Nurses | Training handbook, sessions, epidural policy | Acute Pain Nurse | Acute Pain Nurse, Nurses | Catheter displacement, infusion devices, help-seeking guidance | Not specified | Pain relief (pain scale), safety (adverse events), compliance (protocol adherence), nursing competency |
| Camp-Sorrell | Teaching oncology nurses about epidural catheters. | 1990 | USA | Educate RNs on administering and monitoring epidural analgesia | Pre/ posttest | Nurses | Lecture, practical training, video, protocol | Multidisciplinary team | Clinical Nurse Specialist Pain Team Nurse | Analgesia purpose, patient selection, catheter placement, monitoring, complications, patient education | 4 hours | Knowledge (pre-/post-tests), competency (observed checklist) |
| Cook | Non-Obstetric Safety of Epidurals (NOSE). | 2021 | UK | Improve care standards for perioperative epidural analgesia | Pre-/ Posttest | Nurses Physicians | Formal/informal training, protocol | Multidisciplinary project team | Anaesthetists Clinical Nurse Specialists Lecturer-Practitioners | Patient care, electronic discharges, alert wristbands, motor block checks | Not specified | Knowledge (surveys), skills (motor block checks), confidence, compliance (wristbands, handover), satisfaction |
| Ellis | Evaluation of a continuous epidural analgesia program for postoperative pain in children. | 2007 | Canada | Education and support for safe, effective pain management outside critical care | Descriptive | Nurses  Physicians | Workshops, micro-teaching, interactive sessions, guidelines | Departments of Nursing, Anaesthesia | Clinical Nurse Educator | Pharmacology, sensory assessments, psychomotor skills | Workshop: 4 hours | Pain relief (pain scale). adverse events, programme safety (monitoring) |
| Haas | Standardizing Nursing Assessments of Dermatome Levels of Postoperative Patients with Spinal Blocks, Epidural Analgesia. | 2014 | USA | Standardize dermatome assessments, interventions, and documentation | Pre-/ posttest | Nurses | Formal education, protocol | Education Coordinator | Education Coordinator | Dermatome assessments | 30-min session | Assessment accuracy, protocol knowledge, documentation quality, patient outcomes |
| Ingelmo | Epidural analgesia in children: planning, organization and development of a new program. | 2007 | Argentina | Development and evaluation of a paediatric epidural analgesia programme | Descriptive | Nurses  Physicians  Pharmacists  Midwives | Formal lessons, practical training | Anaesthesia Nurses,  Paediatrics | Anaesthesia Nurses, Paediatrics | Side effect prevention, emergency management, patient communication | Not specified | Pain relief (pain scale), adverse events, ICU stay reduction, safety monitoring |
| Kameni | Distance Learning Education on Labor Epidurals to Nurse Anaesthetists in Cameroon | 2023 | USA | Increase labour epidural knowledge among Cameroonian nurse anaesthetists | Pre-/ posttest | Nurses | Distance learning (videos, podcasts) | Authors | None | Epidural placement and management | 2 months | Knowledge (pre-/ posttests) |
| Kariya | Effects of Simulation Study of High Neuraxial Block During Epidural Analgesia for Labor Pain on Pre/Posttest Evaluation in Junior Clinical Trainees. | 2019 | Japan | Evaluate simulation effects on pre-/post-test performance in junior trainees | Pre-/ posttest | Physicians | Simulation, briefing/ debriefing | Research Team | Board-certified Anaesthesiologist | Technical skills: mask ventilation, sensory block assessment, ephedrine injection | Not specified | Knowledge (pre/ posttest), skills (simulation checklist), response rate |
| Luctar-Flude et al. | Development and evaluation of an epidural analgesia workshop for senior nursing students | 2018 | Canada | Develop and pilot a workshop for senior nursing students using standardized patients | Pre-/ posttest | Nurses | Workshop, online education, pre-workshop guide, lectures, practice scenarios, evaluations | Research Team | Research Team | Preparation, pain/sensory block assessment, motor function, catheter site checks, documentation | Workshop: 4 hours | Knowledge (pre-/posttest), skill performance (checklist), critical thinking (rubric), confidence, satisfaction (feedback) |
| Nowakowski | Implementation of an epidural pain management program. | 1995 | USA | Offer continuous postoperative epidural pain relief for up to three days | Pre-/ posttest | Nurses | Lectures, practical training, guidelines | Anaesthesia, Policy and Procedure Committee, Clinical Nurse Specialist | Critical Care Nurse Specialists | Vital signs, infusion flow rate, respiratory rate, oxygenation, motor strength, infection risks, mental status monitoring | Three-month trial | Pain relief (pain scale), safety (adverse effects), compliance (protocol adherence), competency (monitoring respiratory/motor strength) |
| O’Brian | Developing and implementing a self-learning packet on epidural analgesia. | 1995 | USA | Educate nurses on epidural analgesia using Knowles' principles | Pre-/ posttest | Nurses | Self-learning packet (SLP) | Clinical Nurse Specialist | Clinical Nurse Specialist | Anatomy, physiology, nursing care, pain/motor function assessment | 1 hour | Knowledge (pre-/ posttest), competency (protocol adherence), effectiveness (completion rates, nurse feedback) |
| O'Connor | Success of an interdisciplinary educational program on nursing knowledge about epidural analgesia in a critical care setting | 2015 | UK | Enhance nursing knowledge and improve patient safety by monitoring epidural analgesia | Pre-/ posttest | Nurses | Lectures, Interactive sessions | Research Team | Research Team | Testing/treating block height, recognizing epidural hematoma signs, managing blocks | Not specified | Knowledge (pre-/post-tests) |
| Puthoff | Development of a structured regional analgesia program for postoperative pain management | 2021 | USA | Increase postoperative regional analgesia (RA) usage for NICU surgical patients | Pre-/ posttest | Nurses, Physicians | Lectures, Q&A sessions | Research Team | Research Team | Benefits and safety of RA | Not specified | RA use (percentage), opioid reduction (morphine equivalents), extubation time, safety (hypothermia/complications), satisfaction |
| Richardson | Post-operative epidural analgesia: introducing evidence-based guidelines through an education.... | 2001 | UK | Introduce evidence-based guidelines through education | Pre-/ posttest | Nurses | Lectures, interactive/practical training, patient scenarios | Clinical Nurse Specialist, Lecturer-Practitioner, Anaesthetist | Clinical Nurse Specialist, Lecturer-Practitioner, Anaesthetist | Care planning, epidural guidelines, psychomotor skills | Not specified | Knowledge (pre-/posttest), perceptions (feedback, content analysis) |
| Sawhney | Using Simulation to Enhance Education Regarding Epidural Analgesia for Registered Nurses. | 2018 | Canada | Evaluate simulation-based education on assessing/managing epidural analgesia | Pre-/ posttest | Nurses | Workshop, pre-workshop guide, lectures, practice scenarios, protocol | Interprofessional team of clinical experts | Pain Service Nurse | Anatomy/physiology, pharmacology, complications, sensory/motor blockade, general management | Workshop: 4 hours, Lecture: 1 hour | Knowledge (pre-/posttest), skills (OSCE/checklist), confidence, critical thinking (problem-solving scenarios), satisfaction |
| Sawhney | The Effect of Simulation on Practical Nurses' Assessment of Epidural Analgesia | 2020 | Canada | Assess simulation effectiveness for practical nurses managing epidural analgesia | Pre-/ posttest | Nurses | Workshop, pre-workshop guide, lectures, practice scenarios, protocol | Interprofessional team of clinical experts | Expert Nurse Practitioner | Anatomy/physiology, pharmacology, complications, sensory/motor blockade, general management | Workshop: 4 hours, Lecture: 1 hour | Knowledge (questionnaire), skills (OSCE/checklist), confidence, critical thinking (problem-solving scenarios), satisfaction |
| Zheng | Check the Blood Pressure!: An Educational Tool for Anesthesiology Trainees Converting Epidural Labor Analgesia to Cesarean Delivery Anesthesia. | 2020 | USA | Reduce lapses in hemodynamic monitoring during epidural-to-surgical anaesthesia conversion | Pre-/ posttest | Physicians | Email-circulated practice outline | Research team | Research Team | Hemodynamic monitoring/management during epidural-to-anaesthesia conversion | Not specified | Time gap reduction (<10 min BP intervals), maternal hypotension (SBP <90 mmHg), vasopressor use, fetal outcomes (Apgar scores) |
